# Extracting high-quality RNA from formaldehyde-fixed naturally aged neuromusculoskeletal tissues

**DOI:** 10.2144/btn-2023-0092

**Published:** 2024-02-09

**Authors:** Seth D Thompson, Rajeswari Pichika, Richard L Lieber, Mitra Lavasani

**Affiliations:** 1 Shirley Ryan AbilityLab, Chicago, IL 60611, USA; 2 Department of Physical Medicine & Rehabilitation, Northwestern University, Chicago, IL 60611, USA; 3 Northwestern University Interdepartmental Neuroscience Graduate Program, Northwestern University, Chicago, IL 60611, USA; 4 Edward Hines Jr VA Medical Center, Maywood, IL 60153, USA

**Keywords:** aging, formaldehyde fixation, gene expression, neuromusculoskeletal tissues, RNA isolation from fixed tissue, tissue bank

## Abstract

Modern approaches to discovering molecular mechanisms and validating treatments for age-related neuromusculoskeletal dysfunction typically rely on high-throughput transcriptome analysis. Previously harvested and fixed tissues offer an incredible reservoir of untapped molecular information. However, obtaining RNA from such formaldehyde-fixed neuromusculoskeletal tissues, especially fibrotic aged tissues, is technically challenging and often results in RNA degradation, chemical modification and yield reduction, prohibiting further analysis. Therefore, we developed a protocol to extract high-quality RNA from formaldehyde-fixed brain, cartilage, muscle and peripheral nerve isolated from naturally aged mice. Isolated RNA produced reliable gene expression data comparable to fresh and flash-frozen tissues and was sensitive enough to detect age-related changes, making our protocol valuable to researchers in the field of aging.

## Method summary

This study describes an RNA extraction protocol that successfully produces high-quality RNA from formaldehyde-fixed naturally aged neuromusculoskeletal tissues. Yield, purity and integrity of the RNA were comparable to fresh and flash-frozen tissues isolated from adult and naturally aged mice. This protocol is applicable to a range of tissues, including brain, articular cartilage, skeletal muscle and peripheral nerve, and was validated with subsequent transcriptome analysis.

Continuous advances in high-throughput expression technologies, including multiplex real-time quantitative polymerase chain reaction (qPCR) and next-generation sequencing, have enabled scientists to analyze increasingly smaller quantities of mRNA from a wide range of biological samples [1]. However, studies typically produce this information from fresh or frozen tissues. RNA from fresh tissues can be isolated only at the time of dissection, and many pathology labs are not equipped for frequent flash freezing, mass cryogenic storage or instant tissue processing. For this reason, valuable tissue samples – from animal and clinical studies alike – obtained without immediate plans or capacity for RNA isolation are frequently fixed in formaldehyde, which is a simpler and more time-efficient preservation method. Formaldehyde fixation is generally used to preserve tissue specimens for histological studies and serves as a long-term storage option for tissues with an undetermined purpose. Formaldehyde fixation of pathological or normal tissues preserves tissue structural integrity, but prolonged exposure increases RNA fragmentation and degradation [2]. A reaction among formaldehyde, nucleic acids and surrounding tissue proteins creates methylene bridges [3,4], adding adducts to the RNA and/or bonding it to the surrounding tissue, which can impede RNA isolation and reduce base pairing capabilities. In neuromusculoskeletal tissues isolated from older animals, this impediment is compounded because of increased extracellular matrix deposition and reorganization [5–9], which provides greater protein content for the nucleic acids to bond with during the extraction process when tissues are pulverized. Formaldehyde adducts are in theory reversible [4,10], but previously published protocols for neuromusculoskeletal tissues (Table 1) obtained poor quality RNA, required complex technical requirements (e.g., laser microdissection) or were unable to obtain RNA from samples preserved for longer periods of time. Notably, no previous protocols comparatively assessed their ability to isolate sufficient RNA from aged neuromusculoskeletal tissues. Various aged tissues may also exhibit variability in RNA modification and degradation rates, and the fact that different tissues vary in their relative cellular content and extracellular matrix composition further complicates development of a pan-tissue extraction process.

**Table 1.** Summary of previously published RNA isolation protocols for neuromusculoskeletal tissues.

Model	Tissue	Protocol notes	Fixation	Age	Purification steps	Result	Ref.
Mouse	Cartilage	Laser microdissection; fixed for 3 h; frozen for 20 min	PFA	NS	PK, MgBd	No 260/280 nm or RIN; ∼2 pg of RNA	[11]
Rat	Satellite glial cells	Dissociated to single cells and then fixed for FACS	PFA	10 weeks	FACS, PK, Colm	RIN 7.8	[12]
Human	Brain	Samples were tumors	FFPE	NS	Sec, Colm	1.59–2.03 (260/280 nm); highly degraded; no product >238 bp	[13]
Rat	Brain, cardiac muscle	Automated tissue processor ($15,000); stored for 8 weeks	Formalin, FFPE	NS	Sec, Pulv, Colm	RIN 6.8–6.0; degraded RNA with unremoved methyl groups restricted qPCR; lacks qPCR graphs from formalin samples	[14]
Horse	Brain, muscle	Encephalitis; incubated for 72 h at 56°C; long storage times	FFPE	NS	Sec, PK, Colm	1.65–1.85 (260/280 nm); minimal amplification of products >170 bp; no products >300 bp	[15]
Dog, cat	Brain, muscle	94°C incubation for 10 min; no DNase	FFPE	4, 8, 9 years	Sec, PK	All RIN ≤3.5; no qPCR products >180 bp or from tissues preserved >2 weeks	[16]
Rat, human	Brain	Laser microdissection; applied to microarray	FFPE	4 months, 5 years	PK, Colm	RIN 2.1–3.1; did not test qPCR products >150 bp	[17]

Table shows previously published protocols for isolating RNA from neuromusculoskeletal tissues and highlights issues preventing simple and successful application.

Colm: Column kit purification; FFPE: Formalin-fixed paraffin-embedded; MgBd: Magnetic bead purification; NS: Not stated; PFA: Paraformaldehyde; PK: Proteinase K treatment; Pulv: Tissue pulverization; qPCR: Quantitative polymerase chain reaction; RIN: RNA integrity number; Sec: Section of paraffin-embedded tissues.

A simple and effective protocol to isolate quality RNA from repositories of formaldehyde-fixed aged and diseased neuromusculoskeletal tissues would be of tremendous value, promoting the utilization of powerful transcriptomics technologies for understanding the molecular basis of diseases and, importantly, accelerating the development of potential treatments [18–20]. Therefore, the goal of the present study was to establish a simple method to extract RNA from a range of formaldehyde-fixed tissues – including brain, articular cartilage, skeletal muscle and peripheral nerve – that were isolated from naturally aged mice and stored for up to 1 year.

## Materials & methods

### Materials

#### Supplies

1. 1.5 and 2 ml DNase/RNase-free tubes (022600028; Eppendorf, Hamburg, Germany)
2. TRIzol reagent (15596018; Thermo Fisher Scientific, MA, USA)
3. Proteinase K (AM2542; Thermo Fisher Scientific)
4. SDS (L3771; Sigma-Aldrich, MO, USA)
5. EDTA (E9984; Sigma-Aldrich)
6. Tris-HCl (9310 OP; Sigma-Aldrich)
7. Phosphate-buffered saline (pH 7.2) (14190-144; Thermo Fisher Scientific)
8. RNAlater (AM7020; Thermo Fisher Scientific)
9. Navy RINO RNA Lysis Kit (NAVYR1-RNA; Next Advance, NY, USA)
10. RNeasy Mini Kit (74106; Qiagen, Hilden, Germany)
11. RNase-free DNase set (79254; Qiagen, Hilden, Germany)
12. 80% ethanol (T08204K7; Thermo Fisher Scientific)
13. Chloroform (AC423555000; Thermo Fisher Scientific)
14. Paraformaldehyde (158127-500G; Sigma-Aldrich)
15. Liquid nitrogen

#### Polymerase chain reaction

1. iScript Advanced cDNA Synthesis Kit (1725037; Bio-Rad Laboratories, CA, USA)
2. SsoAdvanced PreAmp Supermix (1725160; Bio-Rad Laboratories)
3. SsoAdvanced Universal SYBR Green Supermix (1725271; Bio-Rad Laboratories)
4. PrimePCR PreAmp Assay for *Gapdh* (10041595; Bio-Rad Laboratories)
5. PrimePCR Assay for *Gapdh* (10025636; Bio-Rad Laboratories)

#### Equipment

1. Centrifuge (5424R; Eppendorf)
2. Ultra-low temperature freezer (U725-86; Eppendorf)
3. Vortex-Genie 2 (SI0236; Scientific Industries, NY, USA)
4. Bullet Blender 24 tissue homogenizer (BBX24B; Next Advance)
5. Spectrum Bessman tissue pulverizer (08-418-3; Thermo Fisher Scientific)
6. Digital dry bath (1660562EDU; Bio-Rad Laboratories)
7. BioSpectrometer Basic with μCuvette (6135000923; Eppendorf)
8. CFX96 Touch Real Time PCR Detection System (1855195; Bio-Rad Laboratories)
9. Walk-in refrigerator (or chromatography refrigerator)

### Methods

#### Tissue collection

This study protocol was reviewed and approved by the Northwestern University Institutional Animal Care and Use Committee. Adult (5 months) and naturally aged (24 months) mice were killed, and whole brain, knee articular cartilage, gastrocnemius muscle and sciatic nerve tissues were collected and stored under three different conditions (n = 3 mice per age per storage condition): 1) formaldehyde-fixed, in which tissues were submerged in formaldehyde (4% paraformaldehyde [w/v] diluted in phosphate-buffered saline) for up to 1 week and then changed to phosphate-buffered saline and stored at 4°C for up to 12 months; 2) fresh, in which tissues were submerged in RNAlater and stored at -80°C overnight; and 3) flash-frozen, in which tissues were snap-frozen in liquid nitrogen and stored at -80°C for 1 week.

### RNA extraction

#### Formaldehyde-fixed tissues

1. Pulverize formaldehyde-fixed tissues in the presence of liquid nitrogen using a Spectrum Bessman tissue pulverizer.
2. Transfer the pulverized tissue into RINO tubes containing Navy beads, add 200 μl of lysis buffer (10 mM Tris-HCl [pH 8.0], 0.1 mM EDTA, 0.2% SDS) and incubate at 80°C in a digital dry bath for 10 min.
3. Reconstitute proteinase K 1 mg/ml in 10% SDS, 50 mM EDTA and 10 mM Tris-HCl (pH 7.4) to make proteinase K buffer.
4. Immediately place samples on ice and add 200 μl of 1 mg/ml proteinase K buffer.
5. Incubate samples for 15 min in a digital dry bath at 56°C.
6. Following incubation, add 1 ml of TRIzol reagent.
7. In a walk-in or chromatography refrigerator, use the Bullet Blender at the maximum speed (dial set to 10) and blend samples for 10 min (because of the settings of the Bullet Blender, this will require two consecutive 5 min blends).
8. Transfer the homogenate to a 2 ml tube and add 300 μl of chloroform.
9. Vortex vigorously for 30 s.
10. Centrifuge the samples at 13,500 relative centrifugal force (RCF) for 15 min at 4°C.
11. Follow steps 8–21 in the following section.

#### Fresh & flash-frozen tissues

1. Thaw the ‘fresh’ sample on wet ice in its enclosed tube until RNAlater is in liquid form and the tissue can be extracted. Flash-frozen samples can proceed directly to step 2.
2. Pulverize tissue samples in the presence of liquid nitrogen using a Spectrum Bessman tissue pulverizer.
3. Transfer pulverized tissues into RINO tubes containing Navy beads with 1 ml TRIzol.
4. In a walk-in or chromatography refrigerator, use the Bullet Blender at the maximum speed (dial set to 10) and blend the samples for 10 min (because of the settings of the Bullet Blender, this will require two consecutive 5 min blends).
5. Transfer the homogenate to a 2 ml tube and add 300 μl of chloroform.
6. Vortex for 5 min and then incubate on ice for 5 min.
7. Centrifuge the samples at 13,500 RCF for 20 min at room temperature.
8. Carefully transfer the aqueous phase (upper clear phase), without disturbing the white interphase or lower pink organic phase, to a separate 2 ml tube.
9. Precipitate RNA from the aqueous phase with 80% ethanol. Invert the tubes carefully to mix the samples.
10. Follow manufacturer instructions for the RNeasy Mini Kit (described immediately below).
11. Add 700 μl of the precipitated RNA to the RNeasy Mini Kit column.
12. Let sit for 5 min.
13. Centrifuge for 1 min at 6000 RCF at room temperature.
14. Discard the flow-through.
15. Add 350 μl of Buffer RW1 (Qiagen) to the column.
16. Centrifuge for 1 min at 6000 RCF at room temperature.
17. Add 80 μl of RNase-free DNase I to the column and incubate at room temperature for 15 min.
18. Add 350 μl of Buffer RW1 to the column and centrifuge at 6000 RCF for 1 min at room temperature.
19. Discard the flow-through, add 700 μl of Buffer RPE (Qiagen) to the column and incubate for 5 min at room temperature.
20. Centrifuge at 6000 RCF for 2 min at room temperature and discard the flow-through.
21. Recover total RNA by adding 30 μl of RNase/DNase-free water.
22. Apply 2 μl of the recovered RNA to a μCuvette and use the BioSpectrometer to measure RNA amount (μg/ml) and purity (260/280-nm ratio).

### cDNA conversion (all samples)

A total of 100 ng of total RNA is reverse transcribed with the iScript Advanced cDNA Synthesis Kit (1 μl of reverse transcriptase and 4 μl of reverse transcriptase buffer, total reaction mixture to 20 μl with RNase/DNase-free water) using the following steps: 42°C for 30 min, 85°C for 5 min and then 4°C indefinitely until ready for use.

### Nonbiased preamplification (fixed samples only)

Prior to amplification of cDNA extracted from formaldehyde-fixed samples, a nonbiased preamplification method is first performed:

1. Mix 25 μl SsoAdvanced PreAmp Supermix with 5 μl preamplification *Gapdh* primer.
2. Add 20 μl of cDNA to the aforementioned mixture.
3. The preamplification reaction is then carried out in a thermal cycler with the following steps: 95°C for 3 min (hot start for Taq polymerase), 95°C for 15 s and 58°C for 4 min. To repeat the second and third steps for 12 cycles.

### qPCR (all samples)

Efficacy of RNA extraction is determined via qPCR for the housekeeping gene *Gapdh*. *Gapdh* is constitutively expressed in all tissues, making it the ideal validation candidate. This final step can be performed for the target gene(s) of choice using the appropriate primers:

1. Dilute cDNA 1:10 in RNase/DNase-free water.
2. Mix 2 μl (fixed) or 1 μl (fresh and flash-frozen) of diluted cDNA, 1 μl of *Gapdh* primers and 10 μl of SsoAdvanced Universal SYBR Green Supermix and bring to a total volume of 25 μl with RNase/DNase-free water.
3. Perform amplification using the following conditions: 95°C for 2 min, 95°C for 5 s and 60°C for 30 s, repeating the second and third steps for 39 more cycles (total of 40 cycles).

## Results & discussion

Total RNA was isolated (n = 3 per age group) from four separate tissues (whole brain, knee articular cartilage, gastrocnemius muscle and sciatic nerve) of adult (5-month-old) and naturally aged (24-month-old) mice using the three distinct preservation methods described earlier (fresh, flash-frozen and formaldehyde-fixed). RNA purity and possible molecular alterations or contaminants were evaluated by measuring the 260/280 nm absorbance ratio. A 260/280 nm ratio ≥1.7 was categorized as sufficient for expression analysis [21]. RNA from all fresh, frozen and fixed samples of both adult and naturally aged tissues yielded RNA with 260/280-nm ratios ≥1.7 (Table 2). These results suggest that our RNA extraction and amplification protocol for fixed tissues produces high-quality RNA regardless of the tissue preservation technique or donor's age. In addition, formaldehyde-fixed tissues from both adult and naturally aged mice (Table 3) yielded total RNA comparable to fresh and flash-frozen samples when normalized to tissue mass.

**Table 2.** The 260/280 nm ratios from adult and naturally aged tissues.

Tissue	Adult			Naturally aged		
	Fresh	Frozen	Fixed	Fresh	Frozen	Fixed
Brain	1.97 ± 0.12	2.05 ± 0.02	1.83 ± 0.14	2.02 ± 0.02	2.05 ± 0.02	1.87 ± 0.06
AC	1.96 ± 0.14	2.01 ± 0.05	1.72 ± 0.10	1.99 ± 0.06	1.84 ± 0.08	1.71 ± 0.01
GM	1.98 ± 0.09	1.94 ± 0.10	1.77 ± 0.15	2.00 ± 0.03	2.02 ± 0.00	1.77 ± 0.21
SN	1.77 ± 0.04	1.75 ± 0.13	1.84 ± 0.11	1.75 ± 0.16	1.98 ± 0.06	1.90 ± 0.17

Table shows the 260/280 nm ratios from total RNA isolated from fresh, frozen and fixed tissues harvested from three independent 5-month-old adult and 24-month-old naturally aged mice. Data are presented as mean ± standard error of the mean (n = 3 per sample treatment).

AC: Articular cartilage; GM: Gastrocnemius muscle; SN: Sciatic nerve.

**Table 3.** Total RNA to initial tissue weight ratio comparison to assess protocol efficacy with adult and naturally aged tissues.

Adult				Naturally aged		
Tissue	Fresh	Frozen	Fixed	Fresh	Frozen	Fixed
Brain	25.3 ± 0.25	57.1 ± 5.38	53.6 ± 3.86	6.78 ± 0.14	9.79 ± 1.03	10.1 ± 0.29
AC	204.7 ± 76.5	525.1 ± 16.5	108.6 ± 20.3	234.5 ± 67.1	168.9 ± 14.3	265.4 ± 43.3
GM	137.4 ± 17.4	39.00 ± 8.0	49.4 ± 13.7	46.6 ± 4.24	13.7 ± 0.80	23.3 ± 6.02
SN	127.9 ± 10.5	19.3 ± 3.9	50.1 ± 17.0	14.9 ± 2.8	12.3 ± 3.26	76.1 ± 14.0

Table shows the ratio of total RNA (ng) to initial tissue weight (mg) at time of isolation in various tissues isolated from three independent 5-month-old adult and 24-month-old naturally aged mice using fresh, frozen and fixed preservation methods. Data are presented as mean ± standard error of the mean (n = 3 per sample treatment).

AC: Articular cartilage; GM: Gastrocnemius muscle; SN: Sciatic nerve.

**Figure 1.**
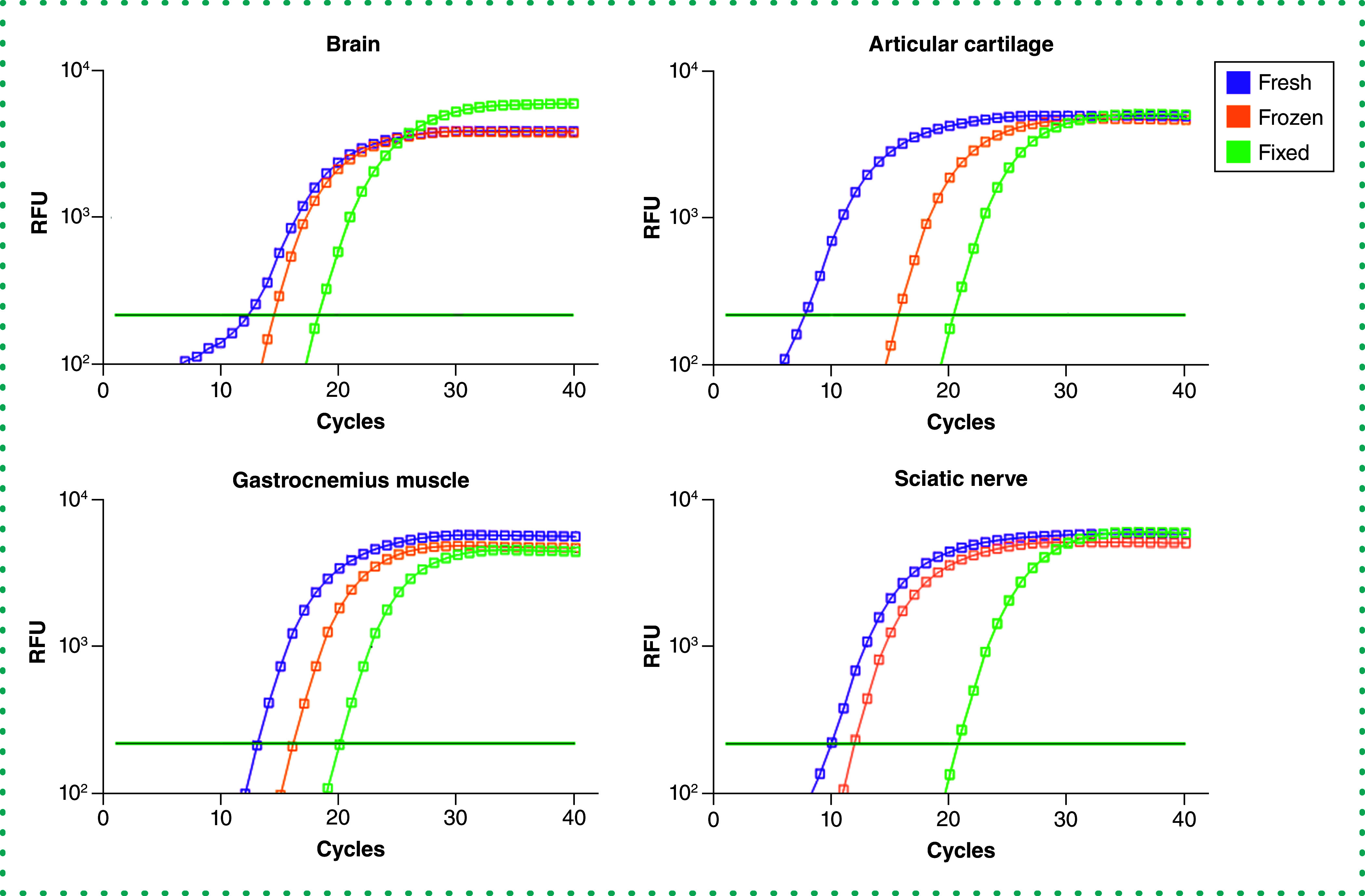
Assessment of amplified mRNA by quantitative polymerase chain reaction. Amplification curves of the housekeeping gene *Gapdh* obtained from naturally aged (24-month-old) mouse brain, articular cartilage, gastrocnemius muscle and sciatic nerve using fresh (purple), flash-frozen (orange) and formaldehyde-fixed (green) preservation methods. Dark green horizontal lines on each graph indicate threshold level. RFU: Relative fluorescence unit.

**Figure 2.**
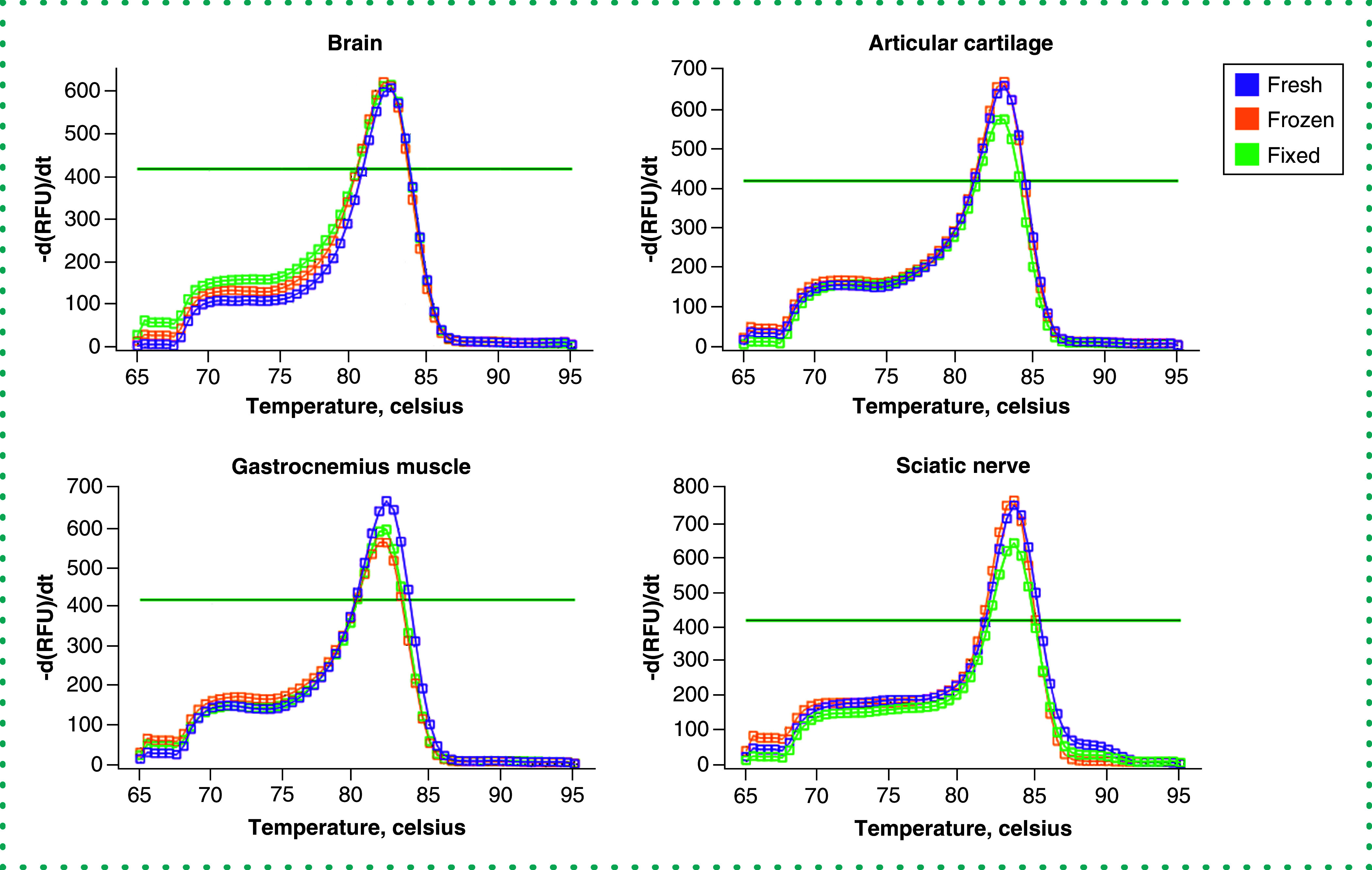
Postamplification melting curves. Graphs showing the melting peaks of the housekeeping gene *Gapdh* from fresh (purple), flash-frozen (orange) and formaldehyde-fixed (green) preservation methods in naturally aged (24-month-old) mouse brain, sciatic nerve, articular cartilage and gastrocnemius tissues. Dark green horizontal lines on each graph indicate threshold level. -d(RFU)/dt: The negative first derivative of change in fluorescence as a function of temperature; RFU: Relative fluorescence unit.

To test our protocol's efficacy, technical duplicates of qPCR were performed targeting the *Gapdh* that was produced using cDNA from each sample. A fluorescence signal above the threshold prior to quantification cycle (C_q_) 35 is considered the minimum acceptable limit. *Gapdh* was detected above the threshold for all samples of the various preservation methods prior to C_q_ 25 (Figure 1). However, both fresh and frozen tissues showed a lower C_q_ value (<20), indicating higher initial copy numbers compared with formaldehyde-fixed tissues (<25 Cq). These amplification curves demonstrate that our protocol produces mRNA of sufficient quality to generate gene expression data from all preservation methods, including formaldehyde-fixed tissues from naturally aged mice. Amplified cDNA was subjected to melting curve analysis to assess the purity of the amplified dsDNA product. Data from all tissues and preservation methods presented a single melting point peak with no discernible differences, demonstrating that each polymerase chain reaction run faithfully amplified the target gene without primer dimerization or incomplete gene products (Figure 2). In addition, RNA isolated from articular cartilage of 2-year-old mice using this protocol was successfully used to perform qPCR analysis on 100% of the 16 targeted genes for gene products up to 182 bp in size [22]. In fact, our recently published results highlight the successful application of this protocol using formaldehyde-fixed aged mouse cartilage, an extremely challenging tissue because of its high proteoglycan content [9,22]. These data affirm that our protocol enables gene expression analysis sensitive enough to detect age-related and tissue rejuvenation changes and that the quality and composition of isolated mRNA are applicable to gene targets beyond the *Gapdh* assay reported here. Together these results demonstrate that our protocol reliably obtains pure mRNA and accurate gene expression products.

Furthermore, our results demonstrate that our protocol has been optimized to efficiently isolate RNA from formaldehyde-fixed tissues isolated from naturally aged mice, as the results are comparable in quality to both fresh and flash-frozen tissues harvested from adult mice. As expected, many aged tissues contained lower initial quantities of RNA; however, we observed that purity was not compromised in aged formaldehyde-fixed samples. Although higher C_q_ values from formaldehyde-fixed tissues following preamplification and qPCR steps indicate a lower initial copy number following the additional RNA extraction steps, mild inhibition of RNA amplification caused by remaining RNA modifications from formaldehyde fixation cannot be ruled out. As previously reported, RNA reconstituted in RNase/DNase-free water rather than Tris buffer presents up to 20% lower 260/280-nm absorbance ratios [21]; therefore, our RNA purity is within the expected range. Notably, since tissue masses were recorded after preservation and each method can substantially alter the tissue's mass, comparisons of RNA yields normalized to tissue mass are more meaningful among tissues of similar preservation methods. In addition, RNA purity was consistent among the various tissues tested and replicates among groups, demonstrating the protocol's reliability and consistency.

As previously mentioned, removal of aldehyde-induced methylene bridges [4,10], which form between nucleic acids and proteins, is important to ‘release’ RNA for isolation and uncover the complete RNA template for amplification. Hence, optimizing the removal of these adducts is a major challenge of RNA purification. Although our method requires heating samples up to 80°C for 10 min, this step is significantly shorter and lower in temperature than the previously reported protocols requiring a 72-h incubation at 56°C [15] and a 10-min incubation at 94°C [16], respectively. The previously reported longer and higher temperature incubations likely cause further RNA degradation and were therefore modified in our protocol. Our shorter and reduced temperature incubation appears to be sufficient to break formaldehyde-generated bonds without producing noticeable RNA degradation.

Using commercially available solutions such as RNase AWAY or RNaseZap (Thermo Fisher Scientific) before and after extraction drastically reduced RNA degradation and potential contamination from ubiquitous work environment RNase. We found that keeping tissue at ice-cold temperatures until RNA was precipitated with 80% alcohol was particularly helpful. However, centrifugation steps were purposefully kept at room temperature for optimal functionality of the column membrane.

As presented, the keys to extracting high-quality mRNA from fixed tissue are extensive tissue pulverization, appropriate time and temperature incubation to reverse adducts and proteinase K treatment to ‘release’ RNA from inappropriate molecular binding. To our knowledge, this is the first detailed report offering a robust methodology to yield quantifiable mRNA from aged neuromusculoskeletal tissues.

## Conclusion

Results from this protocol demonstrate the efficient RNA isolation and amplification and successful gene expression analysis of various formaldehyde-fixed neuromusculoskeletal tissues from both adult and naturally aged mice.

## Future perspective

As high-throughput transcriptomics techniques are becoming prominent in the aging field – which stands to benefit a great deal from making banked tissue samples transcriptomically accessible – our simple, rapid and effective RNA isolation protocol offers a broadly applicable point of entry for basic science and clinical researchers alike. Therefore, utilization of this protocol can streamline access to the genetic information stored within scientifically valuable tissue-banked samples previously preserved in formaldehyde, driving scientific discovery and clinical translation forward.

**Executive summary**

**Background**

- There is a need for a protocol capable of isolating high-quality RNA from difficult-to-process tissues, such as cartilage, particularly when samples are lower in RNA content or more deteriorated, such as in aged animals.

**Materials & methods**

- Brain, articular cartilage, skeletal muscle and peripheral nerve tissues from naturally aged (24-month-old) and adult (5-month-old) mice were harvested as formaldehyde-fixed (and stored for up to 1 year), fresh and flash-frozen samples.
- Fresh and flash-frozen tissues were used as controls.
- Strong tissue pulverization and digestion steps make this protocol simple to perform while maximizing RNA yield from tougher tissues, such as cartilage, without diminishing effectiveness for soft tissues like muscle and brain.

**Results & discussion**

- RNA from formaldehyde-fixed tissues was successfully used for gene expression analysis.
- RNA isolated from formaldehyde-fixed tissues was comparable in quality to control fresh and flash-frozen tissues.
- Single melting point peaks represented faithfully amplified target genes without primer dimerization.

**Conclusion**

- The protocol proved to be simple, fast and effective for isolating quality RNA from a range of formaldehyde-fixed aged neuromusculoskeletal tissues, which has historically been less attainable.
